# Characteristics and spread to the native population of HIV-1 non-B subtypes in two European countries with high migration rate

**DOI:** 10.1186/s12879-015-1217-0

**Published:** 2015-11-16

**Authors:** Kenny Dauwe, Virginie Mortier, Marlies Schauvliege, Annelies Van Den Heuvel, Katrien Fransen, Jean-Yves Servais, Danielle Perez Bercoff, Carole Seguin-Devaux, Chris Verhofstede

**Affiliations:** Aids Reference Laboratory, Department of Clinical Chemistry, Microbiology and Immunology, Ghent University, De Pintelaan 185-Blok A, B-9000, Ghent, Belgium; Aids Reference laboratory, Department of Clinical Sciences, Institute of Tropical Medicine, Nationalestraat 155, B-2000 Antwerp, Belgium; Laboratory of Retrovirology, Department of Infection and Immunity, Luxembourg Institute of Health, Val Fleuri 84, L-1526 Luxembourg, Luxembourg

**Keywords:** HIV-1 non-B infections, HIV-1 subtyping, Subtyping tools, Non-B HIV-1 and co-receptor use, Non-B spread in Western Europe

## Abstract

**Background:**

Non-B subtypes account for at least 50 % of HIV-1 infections diagnosed in Belgium and Luxembourg. They are considered to be acquired through heterosexual contacts and infect primarily individuals of foreign origin. Information on the extent to which non-B subtypes spread to the local population is incomplete.

**Methods:**

*Pol* and *env* gene sequences were collected from 410 non-subtype B infections. Profound subtyping was performed using 5 subtyping tools and sequences of both *pol* and *env*. Demographic information, disease markers (viral load, CD4 count) and viral characteristics (co-receptor tropism) were compared between subtypes. Maximum likelihood phylogenetic trees were constructed and examined for clustering.

**Results:**

The majority of non-B infections were diagnosed in patients originating from Africa (55.8 %), individuals born in Western Europe represented 30.5 %. Heterosexual transmission was the most frequently reported transmission route (79.9 %), MSM transmission accounted for 12.2 % and was significantly more frequently reported for Western Europeans (25.7 % versus 4.3 % for individuals originating from other regions; *p* < 0.001). Subtypes A and C and the circulating recombinant forms CRF01_AE and CRF02_AG were the most represented and were included in the comparative analysis. Native Western Europeans were underrepresented for subtype A (14.5 %) and overrepresented for CRF01_AE (38.6 %). The frequency of MSM transmission was the highest for CRF01_AE (18.2 %) and the lowest for subtype A (0 %). No differences in age, gender, viral load or CD4 count were observed. Prevalence of CXCR4-use differed between subtypes but largely depended on the tropism prediction algorithm applied. Indications for novel intersubtype recombinants were found in 20 patients (6.3 %). Phylogenetic analysis revealed only few and small clusters of local transmission but could document one cluster of CRF02_AG transmission among Belgian MSM.

**Conclusions:**

The extent to which non-B subtypes spread in the native Belgian-Luxembourg population is higher than expected, with 30.5 % of the non-B infections diagnosed in native Western Europeans. These infections resulted from hetero- as well as homosexual transmission. Introduction of non-B variants in the local high at risk population of MSM may lead to new sub-epidemics and/or increased genetic variability and is an evolution that needs to be closely monitored.

## Background

High virus replication rates and error prone reverse transcription resulted in the genetic diversification of the main HIV-1 group M into 9 subtypes: A, B, C, D, F, G, H, J and K [1]. The possibility of recombination further fuels genetic variability. Amongst the constantly increasing number of circulating recombinant forms (CRF), CRF01_AE and CRF02_AG have been the most successful (http://www.hiv.lanl.gov/content/sequence/HIV/CRFs/CRFs.html; last accessed on December 4^th^ 2014) [2]. Between 2004 and 2007, the subtypes C and A, together with the recombinants CRF01_AE and CRF02_AG were responsible for 73 % of all infections worldwide [2]. Since the beginning of the HIV epidemic, non-B infections accounted for up to 50 % of all new diagnoses in Belgium and Luxembourg [3, 4]. Today, the proportion of non-B infections in Luxembourg may reach 62 % [5]. Non-B infections are mainly diagnosed in individuals of foreign origin and in general acquired through heterosexual contact. This contrasts with the epidemic in the local population that is almost exclusively driven by men having sex with men (MSM) infected with subtype B virus. Recent analysis of transmission dynamics in Luxembourg however provided evidence for an increasing number of established non-B transmissions in the country [5]. Subtype specific differences in disease progression, transmission efficiency or susceptibility to antiretroviral drugs have been reported but there still is a lack of consistency in these data [6, 7].

Adequate subtyping is essential when studying the distribution and characteristics of the different non-B subtypes. While the initial classification of HIV-1 into subtypes was based on full length sequences most epidemiological and clinical studies today, for practical reasons, use partial sequences for subtyping. Polymerase (*pol*) gene sequences are preferably used for that purpose because they are widely available through routine drug resistance screening and are considered to contain sufficient phylogenetic signal for subtype assignment [8]. Subtype classification based on one genomic region however, may miss intersubtype recombination [9]. Single region subtyping therefore can introduce bias, especially when studying viral characteristics not defined by the *pol* gene such as co-receptor use. The preference of HIV-1 for one of the two co-receptors, CCR5 and CXCR4, is determined by a small variable region (V3) of the envelope (*env*) gene. The prevalence of CXCR4-use seems to differ between subtypes with low CXCR4-use reported for subtype C and high CXCR4-use for subtype D and CRF01_AE [10–18]. In subtype B, the ability to use CXCR4 has been associated with faster disease progression [19, 20]. For the other subtypes, the potential association between co-receptor use and disease progression is less well documented.

The study presented here is a retrospective analysis comparing the characteristics of non-B HIV-1 infections in two countries with low HIV prevalence but with a high diversity of subtypes [3, 4, 21]. Extended subtyping of 410 non-B infections was performed and patient demographics, HIV transmission route, co-receptor use, viral load and CD4 count were compared between subtypes. The extent to which the non-B subtypes spread in the native population was defined.

## Methods

### Patients and sequences

HIV-1 *pol* sequences (857 nucleotides (nt) long concatenated sequence fragments comprising codon 4 to 99 of the *Protease* gene and codon 30 to 226 of the *Reverse transcriptase* gene), were collected from 410 non-B subtype HIV-1 infected individuals diagnosed at two sites in Belgium (Ghent, 277 patients; Antwerp, 82 patients) and one site in Luxembourg (51 patients) between 2000 and 2012. The *pol* sequences were obtained as part of routine clinical care for baseline or pretreatment resistance analysis. Initial identification of non-B infection was based on the fast subtyping tool implemented in the Smartgene IDNS database system (Smartgene IDNS, Zug, Switzerland). Selected patients had to be treatment naïve and have sufficient left over plasma available for *env* sequencing. *Env* sequences were generated following the procedures for RNA extraction, amplification and Sanger sequencing as described before [22]. This procedure generates an amplicon of about 1106 nt long comprising V1 to V4. The short V3 sequence, with a length of 105 nt, could be generated for all 410 samples but sequencing of a longer *env* fragment (of at least 300 nt) was successful for only 337 samples due to high sequence variability and the abundant presence of indels in this region. Short V3 sequences were used for co-receptor tropism prediction only, the longer sequences were also used for subtype assignment. Demographic, epidemiological and clinical data were collected from the patients’ records, including gender, age, origin, most probable route of transmission, CD4 count and viral load.

### Ethics statement

The study was reviewed and approved by the Ethics Committee of Ghent University Hospital (as central committee; study number 2011/162), the Institute of Tropical Medicine Antwerp and the Comité National d’Ethique pour la Recherche in Luxembourg.

### Subtyping

Subtyping was performed using a combination of 5 subtyping tools: Rega v3, http://regatools.med.kuleuven.be/typing/v3/hiv/typingtool (Rega Institute for Medical Research, Leuven, Belgium) [23]; Comet, http://comet.retrovirology.lu/ (Laboratory of Retrovirology, Luxembourg Institute of Health, Luxembourg) [21]; SCUEAL, http://www.datamonkey.org/dataupload_scueal.php (University of California San Diego, La Jolla, California, US) [24]; jpHMM, http://jphmm.gobics.de/submission_hiv (Institute of Microbiology and Genetics, University of Göttingen, Germany) [25] and NCBI, http://www.ncbi.nlm.nih.gov/projects/genotyping/formpage.cgi (National Center for Biotechnology Information, Bethesda, MD) [26]. To classify *pol* sequences, Comet and Rega were used initially and the subtype was attributed in case of concordant results. Sequences with discordant results or for which the subtype remained unassigned by at least one of the two tools were additionally submitted to SCUEAL and jpHMM. The final subtype was attributed in case of concordance for at least 3 tools. All other sequences were considered as unassigned. The same strategy was used for *env* subtyping but, given that the SCUEAL tool can only handle *pol* sequences it was replaced by NCBI.

### Genotypic tropism prediction

V3 nucleotide sequences were submitted to geno2pheno_(co-receptor)_ (G2P, http://coreceptor.geno2pheno.org, Max Planck Institute for Informatics, Saarbrücken, Germany) for co-receptor tropism prediction [27] and interpreted using the FPR cut off values of 10 and 5.75 %. V3 sequences were also submitted to Web PSSM (http://indra.mullins.microbiol.washington.edu/webpssm/, Department of Microbiology, University of Washington, Seattle, Washington, US) [28, 29] and run with the X4/R5 subtype B matrix and for subtype C also with the subtype C SI/NSI algorithm. For the subtypes A and C and CRF01_AE and CRF02_AG additional tropism prediction was performed with a recently developed subtype specific algorithm, Phenoseq (http://tools.burnet.edu.au/phenoseq/, Burnet Institute, Melbourne, Australia) [30].

### Phylogenetic analysis

*Pol* sequences and *env* sequences >300 bp, were aligned in the BioEdit Sequence Alignment Editor Version 7.0.9. For subtype verification, Los Alamos 2010 subtype and CRF reference sequences with exclusion of the subtype B and group P references (http://www.hiv.lanl.gov/cgi-bin/NEWALIGN/align.cgi; last accessed on December 5^th^ 2014) were added to the alignment. For examination of origin-specific and transmission-route specific clustering the subtype references were excluded. The HXB2 sequence was used as outgroup in all trees. Maximum likelihood trees were generated using the ‘best fit’ evolutionary model selected by jModeltest version 2.1.7 [31] and the PhyML software package with approximate likelihood ratio test (aLRT) for branch support [32]. Tree visualization and editing was done with Itol v2.2.2 [33]. Clusters of presumed recent transmission were identified with the automated cluster selection tool developed recently by Ragonnet-Cronin et al. [34] using as thresholds aLRT >0.9 and mean genetic distance < 0.045.

### Statistical analyses

Statistical analyses were performed using SPSS 22.0 software (IBM Corp. Released 2013. IBM SPSS Statistics for Windows, Version 22.0. Armonk, NY: IBM Corp). Bivariate analyses used the chi-square test or Fisher exact test (if more than 20 % of the cells had expected counts less than 5) for categorical variables and the Mann–Whitney U nonparametric test for continuous variables. The level of significance was set at *p* < 0.05. *P*-values were calculated to assess whether the analyzed parameters showed subtype specific differences. Characteristics of individual subtypes were compared to the pool of all other subtypes.

## Results

### Patients

A total of 410 individuals infected with non-B HIV-1 were selected, 47.3 % were male and the mean age was 33.5 years. Of the 382 individuals with known origin, 213 (55.8 %) were from Africa, 95 (24.9 %) from Belgium or Luxembourg, 22 (5.6 %) from another Western European country, 14 (3.7 %) from Eastern Europe, 32 (8.4 %) from Asia, 5 from South America (1.3 %) and 1 from the Middle East (0.3 %). Of the 303 infections with known infection route, heterosexual transmission was the most frequently reported (242; 79.9 %) followed by MSM transmission (37; 12.2 %), intravenous drug use (IVDU) (10; 3.3 %), blood transfusion (7; 2.3 %) and congenital infection (1; 0.3 %). Multiple risk factors were reported by 6 individuals (2.0 %). Distribution of transmission route for the 184 individuals born outside of Western Europe was heterosexual contact (161; 87.5 %), MSM (8; 4.3 %), blood transfusion (5; 2.7 %), IVDU (5; 2.7 %), congenital transmission (1; 0.5 %) and combined risks (4; 2.2 %). Of the 109 individuals born in Western Europe with known infection route, transmission resulted from heterosexual contact in 73 (67.0 %), MSM in 28 (25.7 %), IVDU in 5 (4.6 %), blood transfusion in 2 (1.8) and combined risks in 1 (0.9 %). The distribution of MSM and heterosexual transmission differed significantly between the individuals born in Western Europe and those born elsewhere (*p* < 0.001).

### Subtyping

Rega and Comet reported a concordant subtype for 351 (85.6 %) of the 410 *pol* sequences. Ten of the 59 sequences with discordant or unassigned subtype could be attributed to a subtype after consulting jpHMM and SCUEAL, 49 (11.3 %) remained unassigned. Subtype distribution was CRF02_AG (100; 27.7 %), A (74; 20.5 %), CRF01_AE (66; 18.3 %), C (63; 17.5 %), G (29; 8.0 %), F (11; 3.0 %), D (8; 2.2 %), CRF06_cpx (4; 1.1 %), CRF12_BF (2; 0.6 %), H (1; 0.3 %), CRF03_AB (1; 0.3 %), CRF11_cpx (1; 0.3 %) and CRF37_cpx (1; 0.3 %). Subtyping of the 318 *env* sequences with Rega and Comet resulted in concordant results for 230 (72.3 %). After consulting jpHMM and NCBI 76 additional sequences were subtyped, 12 (3.8 %) remained unassigned. Because e*nv* does not allow to reliably discriminate between subtype A and CRF02_AG, both were classified as ‘A-like’. Final subtype distribution for the *env* sequences was A-like (148; 48.4 %), C (56; 18.3 %), CRF01_AE (54; 17.6 %), G (21; 6.9 %), B (12; 3.9 %), F (8; 2.6 %), D (6; 2.0 %) and H (1; 0.3 %).

### Concordance between *pol* and *env* subtyping

For comparative analysis of the *pol* and *env* subtyping, *pol* sequences classified as CRF03, CRF06, CRF11, CRF12, CRF13 and CRF37 were equalized to the subtype that constituted the *env* region; respectively B, G, A, F, A and A. Under these conditions, overall concordance between the *pol* and the *env* subtype was 81.1 % (259/318) (Table 1). Lack of concordance resulted from discordant subtype attribution (*n* = 24) or failed subtyping for *pol* (*n* = 23), *env* (*n* = 4) or both (*n* = 8). When the 35 subtyping failures were excluded, overall concordance rose to 91.5 %. Of the 23 samples with unassigned *pol* but assigned *env* subtype, the *env* classification was A-like for 16 (69.5 %), G for 5 (21.7 %) and B for 2 (8.7 %). Twenty four patients (7.5 % of those with both *pol* and *env* subtype available) showed evidence of intersubtype recombination. The novel combinations were CRF02_AG/G (*n* = 4), G/B (*n* = 4), A/B (*n* = 3), D/A (*n* = 3), G/A (*n* = 2), CRF02_AG/B (*n* = 2), CRF01_AE/A (*n* = 1), CRF01_AE/C (*n* = 1), CRF02_AG/D (*n* = 1), A/C (*n* = 1), A/G (*n* = 1) and F/A (*n* = 1). Nine of these potential novel recombinants were classified as subtype B in *env*. Four were identified as CRF14_BG after phylogenetic analysis, 5 were novel non-B/B recombinants. When excluding CRF14_BG, 20 (6.3 %) novel recombinants remained with 6 isolated from native Western Europeans.

**Table 1:** Comparison of the subtypes assigned for the *pol* and *env* sequences

	*Pol* subtype									
*Env* subtype	A	B	C	D	F	G	H	01_AE	02_AG	UA
A-like	**59**			3	1	3		1	**71**	16
B	3	**1**				4			2	2
C	1		**62**					1		
D				**5**					1	
F					**6**					
G	1					**12**			5	5
H							**1**			
CRF01AE								**57**		
UA						2			2	8

Concordant subtypes are marked in bold

*UA*, unassigned

### Phylogenetic and cluster analysis

**Fig. 1:**
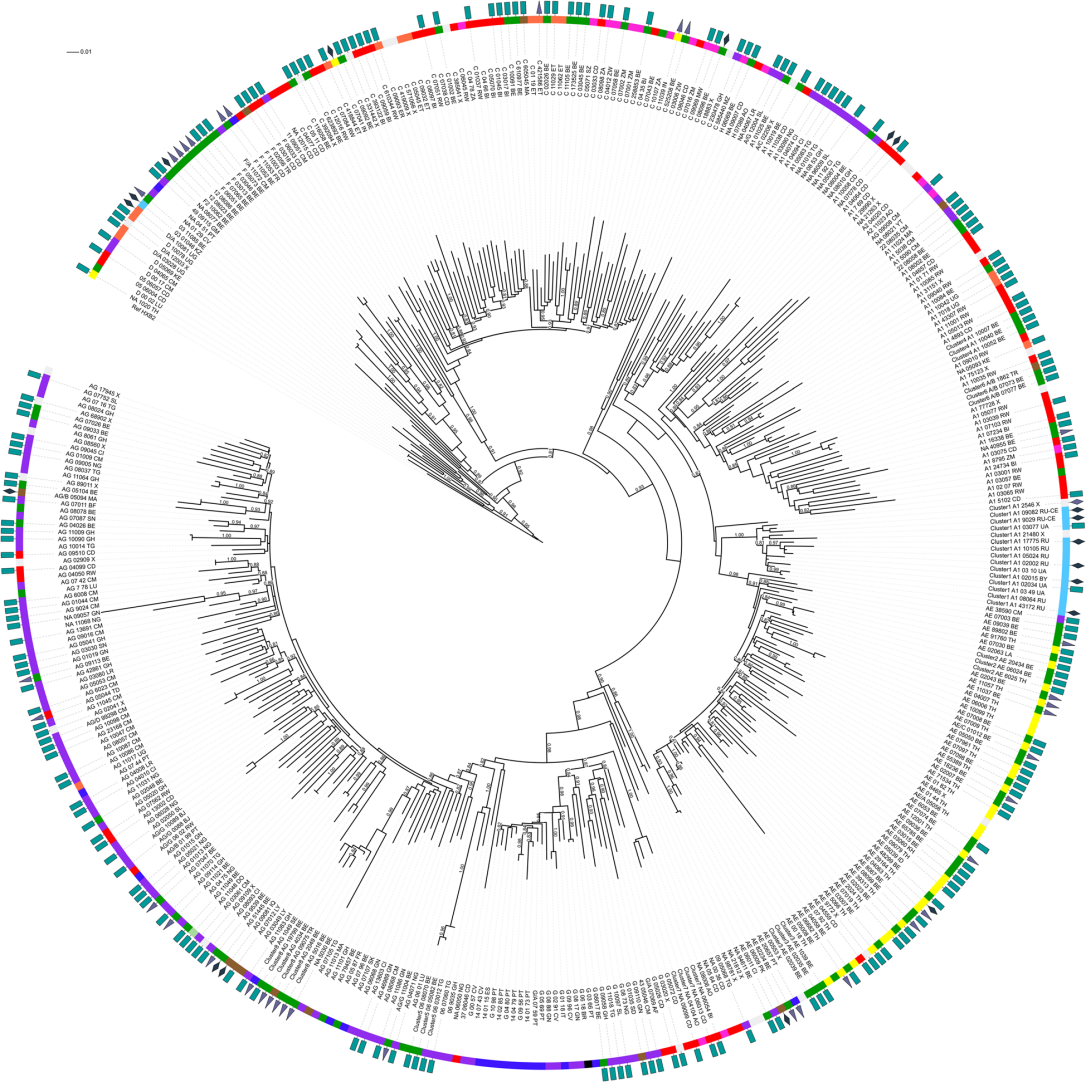
Maximum likelihood phylogenetic tree of HIV-1 *pol* sequences. The HXB2 sequence is used as outgroup, aLRT results are shown if they exceed 0.8. The first characters of the sequence identifier indicate the subtype as assigned by the subtyping tools, the last two characters represent the patients’ country of origin. Specific clustering is also indicated in the sequence identifier (cluster 1 to 8). A color code next to the sequence identifier shows the geographical region of origin of the patient, a symbol indicates the transmission route. Geographical region of origin; Central Africa, red; Eastern Africa, orange; Western Africa, purple; Southern Africa, violet; Northern Africa and the Middle East, brown; Eastern Europe, light blue; Western Europe except Belgium and Luxembourg, dark blue; Belgium and Luxembourg, green; Asia, yellow; South America and the Caribbean, black. Transmission route: rectangle, heterosexual contact; triangle, MSM; diamond, IVDU. Country of origin: Angola, AO; Belarus, BY; Belgium, BE; Benin, BJ; Burkina Faso, BF; Brazil, BR; Burundi, BI; Cambodia, KH; Cameroon, CM; Cape Verde , CV; Congo, CD; Dominican Republic, DO; Eritrea, ER; Ethiopia, ET; France, FR; Gambia, GM; Ghana, GH; Guinea, GN; India, IN; Indonesia, ID; Iraq, IQ; Italy, IT; Ivory Coast, CI; Kazakhstan, KZ; Kenya, KE. Laos, LA; Liberia, LR; Libya, LY; Luxembourg, LU; Malawi, MW; Mayotte, YT, Morocco, MA; Mozambique, MZ; Nigeria, NG; Pakistan, PK; Portugal, PT; Russia, RU; Rwanda, RW; Senegal, SN; Sierra Leone, SL; Slovakia, SK; Sudan, SD; South Africa, ZA; Spain, ES, Swaziland, SZ; Sweden, SE; Thailand, TH; Togo, TG; Chad, TD; Chechnya, RU-CE; Turkey, TR; Uganda, UG; Ukraine, UA; Zambia, ZM; Zimbabwe, ZW

Manual examination of the *pol* phylogenetic tree (Fig. 1) showed marked subtype specific clustering. A large number of the *pol* sequences for which the subtyping tools failed were localized in the subtype A cluster or in the CRF02_AG cluster (respectively 16 and 9 of the 49). Sequences of individuals with Belgian or Luxembourg origin were scattered over the tree. They were however in the majority in the small subtype F cluster. An important representation of Western Europeans, mainly originating from Portugal was also observed for subtype G. In the large and very branched subtype A cluster, a marked clustering (aLRT = 1.0) of sequences from Eastern Europeans, many of whom reporting IVDU transmission, was identified (Fig. 1, cluster 1).

**Fig. 2:**
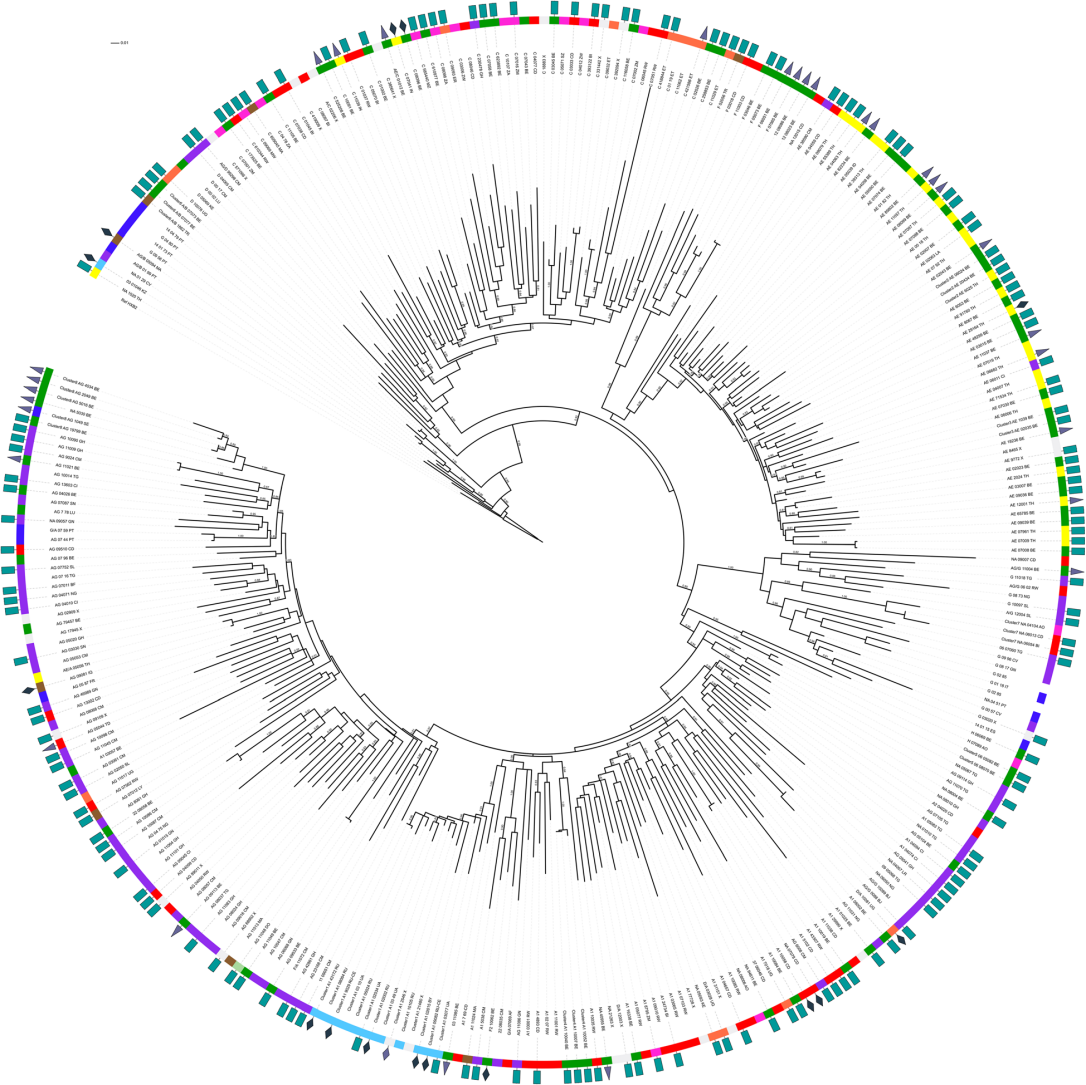
Maximum likelihood phylogenetic tree of HIV-1 env sequences >300 base pairs. The HXB2 sequence is used as outgroup, aLRT results are shown if they exceed 0.8. The first characters of the sequence identifier indicate the subtype as assigned by the subtyping tools, the last two characters represent the patients’ country of origin. Specific clustering is also indicated in the sequence identifier (cluster 1 to 8). A color code next to the sequence identifier shows the geographical region of origin of the patient, a symbol indicates the transmission route. Geographical region of origin; Central Africa, red; Eastern Africa, orange; Western Africa, purple; Southern Africa, violet; Northern Africa and the Middle East, brown; Eastern Europe, light blue; Western Europe except Belgium and Luxembourg, dark blue; Belgium and Luxembourg, green; Asia, yellow; South America and the Caribbean, black. Transmission route: rectangle, heterosexual contact; triangle, MSM; diamond, IVDU. Country of origin: Angola, AO; Belarus, BY; Belgium, BE; Benin, BJ; Burkina Faso, BF; Brazil, BR; Burundi, BI; Cambodia, KH; Cameroon, CM; Cape Verde , CV; Congo, CD; Dominican Republic, DO; Eritrea, ER; Ethiopia, ET; France, FR; Gambia, GM; Ghana, GH; Guinea, GN; India, IN; Indonesia, ID; Iraq, IQ; Italy, IT; Ivory Coast, CI; Kazakhstan, KZ; Kenya, KE. Laos, LA; Liberia, LR; Libya, LY; Luxembourg, LU; Malawi, MW; Mayotte, YT, Morocco, MA; Mozambique, MZ; Nigeria, NG; Pakistan, PK; Portugal, PT; Russia, RU; Rwanda, RW; Senegal, SN; Sierra Leone, SL; Slovakia, SK; Sudan, SD; South Africa, ZA; Spain, ES, Swaziland, SZ; Sweden, SE; Thailand, TH; Togo, TG; Chad, TD; Chechnya, RU-CE; Turkey, TR; Uganda, UG; Ukraine, UA; Zambia, ZM; Zimbabwe, ZW

Using stringent cluster selection criteria (aLRT >0.90, mean genetic distance <0.045 and cluster composed of at least 3 individuals) the *pol* tree was further examined for indications of recent transmission events. Seven transmission clusters were identified; 5 clusters of 3 individuals, 1 of 4 individuals and 1 of 6 individuals. Five of these 7 clusters were also identified in the *env* tree (Fig. 2) although for some the *env* cluster size was smaller because not all individuals had an *env* sequence of >300 bp available. Six of the 7 transmission clusters resulted from heterosexual transmission. They were clusters of CRF01_AE (cluster 2 and 3 in Figs. 1 and 2), subtype A (cluster 4), CRF06_cpx (cluster 5), novel A/B recombinant (cluster 6) and unassigned sequences (cluster 7). The largest cluster (cluster 8) was a CRF02_AG cluster of 6 individuals of whom 5 reported MSM as transmission risk and 5 were of Western European origin.

### Subtype related differences in patient demographics, viral load, CD4 count, co-receptor tropism and transmission route

To ensure sufficient sample numbers for statistical analysis, only the subtypes A and C and the CRF01_AE and CRF02_AG were considered as separate groups, other subtypes and CRF were pooled and sequences with unassigned subtype were excluded. The characteristics of the patients grouped after *pol* subtyping are shown in Table 2. No differences were observed in age or gender distribution, viral load or CD4 counts. Significant differences in the origin of patients were recorded: patients originating from Central and Western Africa and from Eastern Europe were more frequently infected with subtype A, individuals from Western Africa with CRF02_AG, individuals from Asia with CRF01_AE and individuals from Central, East and South Africa with subtype C. Native Western Europeans accounted for between 16.2 % and 47.0 % of all non-B infections. They were underrepresented in subtype A (*p* = 0.008) and overrepresented in CRF01_AE (*p* = 0.001) and in the pooled group of other subtypes and CRFs (*p* = 0.016). MSM as route of transmission was the most frequently recorded for CRF01_AE (18.2 %; *p* = 0.011) but was never reported for subtype A (0 %; *p* = 0.003). IVDU transmission was only reported by patients originating from Eastern Europe and all but one were subtype A infections. CXCR4-using viruses were significantly more prevalent in CRF01_AE (*p* < 0.001) and less prevalent in subtype C (*p* = 0.014) when using G2P or PSSM but not when using Phenoseq. The Phenoseq prediction system on the other hand reported a significantly lower CXCR4-use prevalence in subtype A (*p* = 0.022).

**Table 2:** Patient demographics, viral load, CD4 count, co-receptor tropism and transmission route for the most prevalent subtypes and CRF

		A (*N* = 74)		C (*N* = 63)		CRF_01AE (*N* = 66)		CRF02_AG (*N* = 100)		Other subtypes and CRF (*N* = 63)		*p*
Total, *N* = 366		Count (%)	*p*	Count (%)	*p*	Count (%)	*p*	Count (%)	*p*	Count (%)	*p*	
Gender, *N* = 301												
	Male, N (%)	32 (50)	NS	22 (38.6)	NS	35 (56.5)	0.089	39 (43.3)	NS	13 (46.4)	NS	NS
Age, *N* = 347												
	Median (IQR)	33 (27–40)	NS	34 (27–40)	NS	34 (27–44)	NS	32(28–38)	NS	33 (29–40)	NS	NS
CD4/μl, *N* = 362												
	Median (IQR)	351 (240–567)	NS	310 (146–310)	NS	335 (120–510)	NS	335 (222–535)	NS	293 (141–571)	NS	NS
Viral load, *N* = 363												
	Median log c/ml (IQR)	4.54 (3.85-5.25)	NS	4.35 (3.72-4.86)	NS	4.82 (4.22-5.28)	**0.045**	4.64 (3.88-5.10)	NS	4.51 (3.82-5.00)	NS	NS
Tropisme G2P, *N* = 366												
	X4 (G2P 10 %), N(%)	16 (21.6)	NS	7 (11.1)	**0.014**	30 (45.5)	**<0.001**	17 (17.0)	0.097	14 (22.2)	NS	**<0.001**
	X4 (G2P 5,75 %), N(%)	8 (10.8)	NS	3 (4.8)	**0.014**	24 (36.4)	**<0.001**	9 (9.0)	0.057	10 (15.9)	NS	**<0.001**
Tropisme PSSM, *N* = 360												
	X4 (PSSM-B), N(%)	12 (16.9)	NS	8 (12.7)	0.072	26 (40.0)	**<0.001**	16 (16.3)	NS	14 (22.2)	NS	**0.001**
	X4 (PSSM-C), N(%)			16 (16,3)	NS							
Tropisme Phenoseq, *N* = 277		8 (12.9)	**0.021**	19 (31.1)	NS	13 (21.2)	NS	26 (28.0)	NS			NS
	X4, N(%)											
Transmission route												**0.005**
	Hetero, N (%)	44 (59.5)	NS	37 (58.7)	NS	42 (63.6)	NS	57 (57.0)	NS	39 (61.9)	NS	
	MSM, N (%)	0 (0)	**0.003**	3 (4.8)	NS	12 (18.2)	**0.011**	11 (11.0)	NS	6 (9.5)	NS	
	Other, N (%)	9 (12.2)	**0.020**	2 (3.2)	NS	2 (3.0)	NS	4 (4.0)	NS	5 (7.9)	NS	
	Unknown, N (%)	21 (28,4)	NS	21 (33,3)	NS	10 (15,2)	**0,034**	28 (28,0)	NS	13 (20,6)	NS	
Country of origin, *N* = 366												**<0.001**
	Europe-West, N (%)	12 (16.2)	**0.008**	15 (23.8)	NS	31 (47.0)	**0.001**	22 (22.0)	0.053	27 (42.9)	**0.016**	
	Europe-East, N (%)	13 (17.6)	**<0.001**	0 (0)	NS	0 (0)	0.082	0 (0)	**0.014**	1 (2.4)	NS	
	Africa-Nord/Middle East, N (%)	2 (2.7)	NS	1 (1.6)	NS	0 (0)	NS	4 (4.0)	NS	1 (1.6)	NS	
	Africa-West, N (%)	10 (13.5)	**0.016**	1 (1.6)	**<0.004**	2 (3.0)	**<0.001**	58 (58.0)	**<0.001**	20 (31.7)	NS	
	Africa-Central, N (%)	26 (35.1)	**<0.001**	19 (30.2)	**0.001**	1 (1.5)	**<0.001**	7 (7.0)	**0.003**	7 (11.1)	NS	
	Africa-East, N (%)	2 (2.7)	NS	8 (12.7)	**0.002**	0 (0)	**0.050**	2 (2.2)	NS	4 (6.3)	NS	
	Africa-South, N (%)	2 (2.7)	NS	11 (17.5)	**<0.001**	0 (0)	NS	0 (0)	**0.023**	0 (0)	NS	
	Asia, N (%)	0 (0)	**0.004**	2 (3.2)	NS	29 (43.9)	**<0.001**	0 (0)	**<0.001**	0 (0)	**0.007**	
	Latin America, N (%)	0 (0)	NS	0 (0)	NS	0 (0)	NS	1 (1)	NS	1 (1.6)	NS	
	Unknown, N (%)	7 (9.5)	NS	6 (9.5)	NS	3 (4.5)	NS	6 (6.0)	NS	2 (3.2)	NS	

Subtype classification based on *pol* sequences

Significance was set at ≤0,05; *p*-values ≤0,1 are shown; *p*-values of ≤0,05 are marked in bold; NS = not significant, *p* > 0,1

The analysis presented in Table 2 was repeated after grouping the patients based on *env* subtyping. The outcome was comparable (results not shown).

### Characteristics of the non-B infections in the native population

The majority (91.6 %) of the Western-Europeans in the cohort were of Belgian or Luxembourg origin. The subtype distribution amongst this native population was CRF01_AE (30.6 %), CRF02_AG (19.4 %), C (15.3 %), A (12.2 %), F (8.2 %), CRF06 (2.0 %), D (1 %), G (1%), H (1 %), and unassigned (9.2 %). The main infection route was heterosexual (68.1 %) followed by MSM (28.6 %) and blood transfusion (3.3 %).

## Discussion

Several Western European countries, especially those with large sub-Saharan African immigrant communities, have a high burden of HIV-1 non-B infections. The vast majority of these infections are acquired through heterosexual contacts. This contrasts with the subtype B epidemic in the same countries, that is driven predominantly by MSM and affects primarily young white men [35, 36]. Increasing frequencies of non-B infections over time have been reported for Belgium [4], Spain [37], Italy [38], France [39], Sweden [40, 41] and Luxembourg [21]. Although information on the extent to which the subtype B and non-B epidemics intermingle remains limited, ascending prevalence’s of non-B subtypes in native Europeans have been reported [21, 37, 41].

For this study we selected 3 sites in Belgium and Luxembourg, two small countries with a high migration rate, for investigation of the non-B infections. Although the study is biased by the random selection of non-B infections and the unequal distribution of patients across the territory and can therefore not be seen as representative for the general epidemic in these countries, it does provide some interesting insights in the dynamics of the non-B infections. Important strengths of the study are the large sample size, the high subtype and CRF heterogeneity and the availability of both *pol* and *env* sequences for subtype assignment.

A higher than expected percentage of patients with non-B infections were of Western European origin (30.7 %; 24.5 % from Belgium or Luxembourg and 5.6 % from other Western European countries). While heterosexual transmission accounted for the majority (79.3 %) of infections a significant proportion of the patients reported MSM transmission (13.3 %). MSM infections were more frequent in Western Europeans (28.6 % versus 5.1 % non-Western Europeans; *p* < 0.001) providing proof for the spread of non-B subtypes to the native high-at-risk MSM population. One MSM transmission cluster of CRF02_AG was identified but this was the only clear phylogenetic indication of local non-B transmission in MSM. CRF01_AE was the most represented non-B subtype in Western Europeans (38.6 %), followed by CRF02_AG (27.7 %), C (19.3 %) and A (14.5 %). The higher prevalence of CRF01_AE in individuals born in Western Europe can have several reasons. The Philippines and Thailand, where CRF01_AE is the major subtype [42] are in the top 5 of bride-donor-countries of Belgium (http://www.esf-agentschap.be/sites/default/files/attachments/articles/eindrapport_partnermigratie.pdf). Sex tourism to South-East Asia may also have triggered the presence of CRF01_AE, in heterosexuals as well as MSM. MSM were significantly more represented amongst the CRF01_AE infections compared to the other subtypes. The profound intermingling of the CRF01_AE sequences from native Western Europeans and Asians and the lack of clear indications for onward local CRF01_AE transmission apart from some paired transmissions suggest that most CRF01_AE infections are imported infections.

Infection through IVDU was reported infrequently and only in Eastern-European individuals. The phylogenetic analysis revealed a very close genetic relationship between the viruses isolated from these Eastern-European IVDU but no indications for a spread of these particular strains to the local population.

Weakening of the association between subtypes and patient origin as well as between subtypes and exposure group has been reported before for the UK, Spain, Italy and Sweden [41, 43–46]. Future surveillance of this evolution will be important because this tendency can have implications on many facets of HIV care and prevention. Introduction of non-B subtypes in native high-at-risk populations may increase the overall genetic heterogeneity and the frequency of intersubtype recombination. Recombination may occasionally result in viral variants with increased pathogenesis as demonstrated recently in Cuba [47]. In our study, indications for novel intersubtype recombination were found in 6.3 % of the patients and a high contribution of non-B/B recombinants was noticed (5 of the 20 recombinants). This number of novel non-B/B recombinants is almost certainly an underestimation as the initial selection of non-B infections was based on the *pol* subtype and excluded infections classified as subtype B in *pol*. High percentages of novel recombinants have been reported for Spain (13.4 %) [48], Italy (9.4) [38] and UK (9.9 %) [49]. In these studies the classification of novel recombination is based only on the *pol* sequence. In our study however, 25 of the 49 *pol* sequences considered as having a complex mosaic genetic pattern, were assigned A-like in *env* and classified as subtype A by *pol* phylogenetic analysis. Adequate classification of subtype A and CRF02_AG *pol* sequences seems particularly challenging because of the overall high genetic variability within these subtypes. This apparently may lead to a false identification of novel recombinants and may partly explain the important differences in frequency of novel recombination between studies, a hypothesis that needs to be confirmed. Local spread of these novel recombinants was still limited, only one small transmission cluster of a novel A/B recombinant was observed.

The patients infected with the most represented subtypes, A, C, CRF01_AE and CRF02_AG, did not differ in age, gender or CD4 count. The significant differences in distribution of patient origin followed the geographic distribution pattern of the respective subtypes [2].

The higher prevalence of CXCR4-use in CRF01_AE when using the G2P or PSSM prediction algorithms and the lower prevalence of CXCR4-use in subtype C confirmed previous findings [10, 11, 14, 50, 51]. It is known however that G2P and PSSM lack accuracy for non-B subtypes [14, 52, 53]. Recently, an algorithm has been developed that claims high sensitivity and specificity for the subtypes A, B, C, D, CRF01_AE and CRF02_AG [30]. When applying this algorithm the prevalences of CXCR4-use for the subtypes A and CRF01_AE differed considerably from the ones recorded with G2P and PSSM. These observations show that problems with co-receptor tropism prediction of some non-B subtypes are still unresolved and that urgent actions for improvements are needed.

We were unable to define subtype related differences in viral load or CD4 count but have to acknowledge that it is a shortcoming of this study that viral load and CD4 data were based on single measurement and could not be corrected for time of infection.

## Conclusion

A higher than expected proportion of the non-B infections sampled in Belgium and Luxembourg were diagnosed in individuals of Western European origin. The spread of African and Asian subtypes to the local population resulted from heterosexual as well as MSM transmission. Local spread remains limited. Subtype related differences in patient origin, infection route and CXCR4-use were noticed but no differences in gender, age, CD4 count or viral load were found. Future monitoring of the introduction of non-B infections in the local population is warranted both from an epidemiological and a prevention perspective.

### Availability of supporting data

Sequences were submitted to the National Center for Biotechnology Information (NCBI) Genbank and are available under accession numbers KT863537 to KT863946 (protease sequences), KT863947 to KT864356 (reverse transcriptase sequences) and KT864357 to KT864674 (envelope sequences).

## Abbreviations

HIV: Human Immunodeficiency Virus
CCR5: Chemokine (C-C motif) Receptor type 5
CXCR4: Chemokine (C-X-C motif) Receptor type 4
Pol: Polymerase
Env: Envelope
MSM: Men having sex with men
CRF: Circulating recombinant from
Nt: Nucleotide
IDNS: Integrated database network system
V3: Variable region 3
PSSM: Position specific scoring matrix
G2P: Geno-to-Pheno
SCUEAL: Subtype classification using evolutionary algorithms
jpHMM: Jumping profile hidden markov model
NCBI: National center for biotechnology
aLRT: Approximate Likelihood Ratio Test
PhyML: Maximum likelihood phylogenies
SPSS: Statistical package for the social sciences
IBM: International business machines corporation
IVDU: Intravenous drug use
